# Multicenter phase II study of weekly docetaxel, cisplatin, and S-1 (TPS) induction chemotherapy for locally advanced squamous cell cancer of the head and neck

**DOI:** 10.1186/1471-2407-13-102

**Published:** 2013-03-06

**Authors:** Woo Kyun Bae, Jun Eul Hwang, Hyun Jeong Shim, Sang Hee Cho, Ki Hyeong Lee, Hye Suk Han, Eun-Kee Song, Hwan Jung Yun, In Sung Cho, Joon Kyoo Lee, Sang-Chul Lim, Woong-Ki Chung, Ik-Joo Chung

**Affiliations:** 1 Department of Hematology-Oncology, Chonnam National University, Gwangju, Korea; 2 Department of Hematology-Oncology, Chungbuk National University, Cheongju, Korea; 3 Department of Hematology-Oncology, Chonbuk National University, Jeonju, Korea; 4 Department of Hematology-Oncology, Chungnam National University, Daejeon, Korea; 5 Eulji University Hospital, Daejeon, Korea; 6 Department of Otorhinolaryngology-Head and Neck Surgery, Chonnam National University, Gwangju, Korea; 7 Department of Radiation Oncology, Chonnam National University, Gwangju, Korea; 8 Department of Internal Medicine, Chonnam National University Hwasun Hospital, 160 Ilsim-ri, Hwasun-eup, Hwasun-gun, 519-809, Korea

**Keywords:** Head and neck squamous cell carcinoma, Induction chemotherapy, Cisplatin-based regimen containing S-1, Weekly docetaxel

## Abstract

**Background:**

The purpose of this study was to evaluate the efficacy and tolerability of weekly docetaxel, cisplatin, and S-1 (weekly TPS) as induction chemotherapy for patients with locally advanced head and neck squamous cell carcinoma (HNSCC).

**Methods:**

A total of 35 patients with previously untreated, locally advanced HNSCC were enrolled. Seven patients (20%) were diagnosed with stage III HNSCC and 28 patients (80%) were diagnosed with stage IV. Induction treatment included 30 mg/m^2^ docetaxel on day 1 and 8, 60 mg/m^2^ cisplatin on day 1, and 70 mg/m^2^ S-1 on days 1 to 14. The regimen was repeated every 21 days. After three courses of induction chemotherapy, patients received concurrent chemoradiotherapy.

**Results:**

Among the 35 patients, 30 (85.7%) completed induction chemotherapy. The response to induction chemotherapy was as follows: nine patients (25.7%) achieved a complete response (CR) and the overall response rate (ORR) was 85.7%. Grades 3–4 toxicity during induction therapy included neutropenia (28.5%), neutropenic fever (8.5%), and diarrhea (17.1%). After completion of concurrent chemoradiotherapy, the CR rate was 62.8% and the partial response (PR) was 22.8%. Estimates of progression-free and overall survival at 2 years were 73.2% and 79.3%, respectively.

**Conclusions:**

Weekly TPS is a promising regimen that is well-tolerated, causes minimal myelosuppression and is effective as an outpatient regimen for locally advanced HNSCC.

**Trial registration:**

ClinicalTrials.gov: NCT01645748

## Background

Head and neck squamous cell carcinoma (HNSCC) ranks sixth among the most common cancers, accounting for approximately 5% of all cases of cancer [1]. The disease is potentially curable at an early stage, but 40% to 50% of patients present with locally advanced disease [2]. Meta-analysis of adjuvant chemotherapy in head and neck cancer suggests an increase absolute survival [3-5]. Combined modality approaches have been developed in an effort to enhance loco-regional disease control, reduce distant metastatic spread, and improve survival in patients with inoperable head and neck cancer [6-8].

Contrary to the usual pattern of failure, several studies have shown that in patients treated with CCRT, there was an increase in systemic relapse due to a lack of systemic control [9,10]. In this regard, interest in the use of induction chemotherapy has been shown. The use of induction chemotherapy is based on two hypotheses. One involves the better delivery of the drug in untreated, well-vascularized tumors, and the second involves the eradication of the micrometastatic disease with systemically active doses of chemotherapy [11]. In addition, a patient who is treatment-naïve may be more tolerant of the adverse effects of chemotherapy treatment compared with a patient who has received prior radiation [12].

Even though several meta-analyses failed to reveal any significant improvement in survival using adjuvant chemotherapy [4,13], two phase II trials have explored and highlighted the role of induction chemotherapy using three-drug combination chemotherapy regimens comprising fluorouracil, cisplatin, and a taxane [14,15]. Subsequently, phase III trials of a TPF regimen (using docetaxel) significantly improved the rate of progression and overall survival not only in patients with unresectable HNSCC [16], but also in localized HNSCC followed by CCRT [17]. We also previously reported a 95.4% overall response rate and 88.7% progression-free survival at 2 years in a phase II study using TPF induction chemotherapy in locally advanced HNSCC [18].

Therefore, the TPF regimen has become the standard treatment for induction or palliative treatment of advanced HNSCC. However, the TPF regimen has been shown to cause severe myelotoxicity (*e*.*g*., grades 3–4 neutropenia in 80–90% of patients). Docetaxel is widely used to treat solid tumors such as breast cancer, lung cancer, gastric cancer and esophageal cancer. To reduce the associated toxicity, several studies have suggested that weekly doses of docetaxel may help reduce bone marrow suppression relative to 3-week regimens, which show similar efficacy [19-21]. However, there has been no report of use of these treatment regimens in head and neck cancer patients.

S-1 is an oral fluoropyrimidine derivative that was developed in Japan, based on the concept of biochemical modulation. It consists of the following three components in a molar ratio of 1:0.4:1; tegafur, a prodrug that is slowly metabolized to 5-fluorouracil; gimeracil, which reversibly inhibits dihydropyrimidine dehydrogenase, the rate-limiting 5-fluorouracil-degrading enzyme, use of which results in an increase in the plasma concentration of 5-fluorouracil; and oteracil potassium, which is distributed in high concentrations in gastrointestinal tissue and inhibits phosphorylation of 5-fluorouracil, reducing its gastrointestinal toxicity. S-1 was developed to achieve enhanced efficacy with lower toxicity when compared to conventional 5-fluorouracil derivatives [22]. The oral administration of S-1 enables chronic daily dosing and results in effects similar to continuous 5-fluorouracil infusion without the complications and inconvenience associated with central venous catheter access and S-1 offers the potential for more convenient outpatient treatment.

Therefore, this study was conducted to evaluate the efficacy and tolerability of weekly docetaxel and TS-1 compared to 3-week docetaxel and intravenous 5-FU treatment, with cisplatin as an induction chemotherapy, for locally advanced HNSCC.

## Methods

### Patients

Patients were eligible if they had locally advanced stage III or IV squamous cell carcinoma of the larynx, oropharynx, or hypopharynx. Patients at least ≥18 years old with adequate bone marrow and organ function were included in this study (*i*.*e*., absolute neutrophil count ≥1,500/μL, platelets ≥100,000/μL, serum bilirubin <2.0 mg/dL, creatinine <1.5 mg/dL, and serum transaminase levels less than twice the upper limit of normal). Patients were excluded from the study if they had received previous chemotherapy. Other exclusion criteria included history of another malignancy; pregnancy or lactation; current or history of distant metastasis; history of clinically significant cardiac disease (serious arrhythmia, heart failure, myocardial infarction, or unstable angina) within the last 6 months; active serious infection; or a psychiatric illness that would preclude obtaining informed consent. Patients with nasopharyngeal carcinoma were also excluded from the study.

Pretreatment staging involved examination of the ears, nose, and throat by an otolaryngologist, as well as a computed tomographic (CT) scan or magnetic resonance imaging (MRI) of the primary tumor site and neck. To detect other primary aerodigestive tract malignancies, patients underwent a CT scan of the chest and an esophagogastroduodenoscopy or pharyngoesophagram. Before radiation therapy, all patients received a dental examination to avoid unexpected osteonecrosis or osteomyelitis associated with radiation.

All patients provided written informed consent before being enrolled in the study, which was approved by the institutional review board of each participating hospital.

### Induction chemotherapy

Docetaxel (30 mg/m^2^) was given as a 1-h intravenous infusion on days 1 and 8. Cisplatin (60 mg/m^2^) was given as a 3-h intravenous infusion on day 1. S-1 was given orally twice daily, at 70 mg/m^2^, for 14 consecutive days. The cycles were repeated every 3 weeks. Patients received further cycles of chemotherapy only when the absolute neutrophil count was ≥1,000/mm^3^ and the platelet count was ≥100,000/mm^3^. Toxicity was graded according to the National Cancer Institute Common Toxicity Criteria (NCI-CTC), version 3.0. Dose modifications were determined based on hematological and non-hematological toxicity. The dose of docetaxel was reduced by 20% after any episode of febrile neutropenia or grade 4 neutropenia lasting more than 5 days or for grade 4 thrombocytopenia and grade 3 or 4 non-hematological toxicity, except alopecia. If these complications developed after docetaxel dose adjustment, the S-1 dose in the next cycle was reduced by 20%. The dose of docetaxel on day 8 was interrupted in cases of grade 2 or higher neutropenia and thrombocytopenia and postponed to day 10. Docetaxel treatment on day 10 was withheld in cases of grade 4 neutropenia and grade 3 or 4 thrombocytopenia. All patients received pre- and post-cisplatin intravenous hydration. Standard intravenous premedications with dexamethasone, diphenhydramine, and ranitidine were administered 30 min before docetaxel infusion to prevent hypersensitivity reactions. A prophylactic antibiotic (levofloxacin 500 mg) was given orally for 5 days of each chemotherapy cycle. As supportive treatment for grade 4 neutropenia and grade 3 or 4 febrile neutropenia, granulocyte colony-stimulating factor (G-CSF) and antibiotics were given at the investigators’ discretion.

### Concurrent chemoradiotherapy

After three cycles of induction chemotherapy, patients had radiotherapy using 6 MV photon beams produced by linear accelerator having multi-leaf collimator. All patients had CT scanning for radiation treatment planning at supine position with a thermoplast immobilization mask. Primary tumor site and upper neck node bearing areas were irradiated by two parallel opposing fields with half beam technique, and lower neck and supraclavicular nodes by matched anterior one field. The spinal cord was shielded after 44 or 45 Gy, and then treatment fields were gradually reduced so that the volume containing the gross disease was irradiated with a curative dose. All patients were treated with standard radiotherapy technique in daily fraction of 1.8 or 2 Gy, 5 days per week. The gross neck node had a boost irradiation with a 9 MeV or 12 MeV electron beam. The primary tumor site and gross neck node area received 65 to 70 Gy over 7 to 8 weeks. A minimum of 45 Gy was delivered to clinically uninvolved neck nodes and supraclavicular nodes.

Definite irradiation was scheduled with concurrent administration of cisplatin in all patients except for those whose performance status or residual toxicities precluded the co-administration of chemotherapy. Cisplatin was given every 3 weeks at a dose of 100 mg/m^2^, depending on creatinine clearance. Doses of cisplatin were delayed if there was evidence of dehydration, renal toxicity, neurotoxicity or ototoxicity. For patients with grades 3–4 mucositis or dysphagia, radiation therapy was delayed until recovery to less than grade 2 toxicities.

### Follow-up and evaluation

After three cycles of induction chemotherapy and 6–8 weeks after completion of CCRT, clinical responses were assessed. Patients underwent examination by an otolaryngologist, as well as CT or MRI imaging of the primary tumor and neck. A biopsy of the primary site was recommended if possible. Tumor response was assessed according to the Response Evaluation Criteria in Solid Tumors (RECIST). For all patients with a complete response (CR) on the physical examination and CT or MRI scans, an [^18^ F] fluorodeoxyglucose positron emission tomography (^18^ F-FDG-PET) scan was performed as a confirmation at 3 months after CCRT. When CCRT was completed, patients were observed on a monthly basis by a physician examination to evaluate the status of the disease and toxicity; CT or MRI scanning was performed every 3 months until disease progression. Toxicity was assessed according to the NCI-CTC, version 3.0. Quality of life (QOL) measures were assessed using the European Organization for Research and Treatment of Cancer Core Quality of Life questionnaire (EORTC QLQ C-30 version 3) at baseline and after three cycles of induction chemotherapy. During radiation therapy, the toxicities were evaluated by a physician every 2 weeks.

The dose intensity was calculated as the ratio of the total dose per square meter of body surface area divided by the total treatment duration (presented as mg/m^2^/week). In this calculation, the end of treatment was considered to be 21 days after day 1 of the last cycle of chemotherapy. The relative dose intensity was calculated as the ratio of the dose intensity actually delivered to that planned.

### Statistical analysis

The primary endpoint was objective response, and secondary endpoints were safety, QOL, progression-free survival (PFS), and overall survival (OS). The study was conducted using a Simon’s two-stage phase II design. A sample size of 35 patients was required to accept the hypothesis that the true response rate was greater than 90% with an 85% power and to reject the hypothesis that the response rate was less than 70% with 5% significance. Initially we planned to enroll 18 patients in the first stage. If 13 or more responses were observed, we planned to continue to the second stage for a total of 32 patients in the analysis. Assuming a dropout rate of 10%, the total number of enrolled patients needed was calculated to be 35. The OS was measured from the start of chemotherapy until the date of death or the last confirmed date of survival. The PFS was defined as the time from the start of chemotherapy to the first appearance of progressive disease or death from any cause. The survival analysis was performed using the SPSS software (version 18.0; SPSS Inc., Chicago, IL, USA), and 95% confidence intervals (CIs) were calculated for all relevant estimates using StatXact (version 8; Cytel, Cambridge, MA, USA).

## Results

### Patient characteristics

Based on the previously described Simon’s two-stage design, 18 patients who met the criteria were enrolled, and 15 patients (83.3%) achieved an objective response rate (ORR). Therefore, the study proceeded to the next stage, and a total of 35 patients were enrolled.

Patient baseline characteristics are given in Table 1. Thirty-five patients with locally advanced HNSCC were enrolled between October 2008 and October 2011 as the intention-to-treat (ITT) population. The enrolled population consisted of 33 males and two females, with a median age of 57 years (range, 29–72 years). Most patients (80%) had stage IV cancer; 20% had stage III HNSCC. The most common primary tumor site was the oropharynx (48.6%), followed by the hypopharynx (28.6%) and the larynx (22.9%). All 17 patients with oropharyngeal cancer were smokers. Twelve of these patients had evaluable specimens, none of which were positive for human papillomavirus (HPV) DNA by *in situ* hybridization (ISH).

**Table 1:** Patient and disease characteristics

**Characteristics**	**Number (%)**
Total patients	35
Age (years)	
Median ± SD	57.4 ± 9.5
Sex	
Male	33 (94.3)
Female	2 (5.7)
ECOG Performance status	
0	17 (48.6)
1	17 (48.6)
2	1 (2.9)
Tumor (T)	
T1	3 (8.6)
T2	16 (45.7)
T3	9 (25.7)
T4	7 (20.0)
Lymph node (N)	
N0	3 (8.6)
N1	6 (17.1)
N2	24 (68.6)
N3	2 (5.7)
AJCC/UICC staging system	
III	7 (20.0)
IVA	25 (71.4)
IVB	3 (8.6)
Primary tumor site	
Oropharnyx	17 (48.6)
Hypopharynx	10 (28.6)
Larynx	8 (22.9)

### Treatment

A total of 97 cycles of weekly TPS therapy were given to 35 patients, and 30 completed the scheduled CCRT. Three patients received only one cycle and two patients received two cycles of TPS due to toxicological effects. The median duration from day 1 of cycle 1 to day 1 of cycle 3 of induction chemotherapy was 6.6 weeks (range, 5.8–9.4 weeks), and the median duration from day 1 of cycle 3 of chemotherapy to day 1 of CCRT was 5.3 weeks (range, 2.5–11.2 weeks). The mean dose intensities relative to the target dose of docetaxel, cisplatin, and TS-1 were 97.7%, 98.6%, and 97.7%, respectively. The most common reasons for a decrease in dose intensity were neutropenia and diarrhea. During CCRT, the mean dose intensities relative to the target dose of cisplatin was 69.6%. RT was interrupted in 10 patients (33.3%); of these, six had grades 3–4 mucositis, two had grade 2 renal toxicity, and two had grade 3 fatigue. However, RT was continued and completed in these patients after no more than 2 weeks of rest.

### Response and survival

Thirty patients (85.7%) were evaluable for a response. The five patients that were not evaluable were included in the ITT analysis and kept in the denominator for calculation of the response rate. Five patients discontinued induction chemotherapy; three due to adverse events (grade 4 neutropenia or febrile neutropenia), one was lost to follow-up (this patient showed a near clinical CR), and one died of asphyxia associated with vocal cord paralysis due to his oropharyngeal cancer.

**Figure 1:**
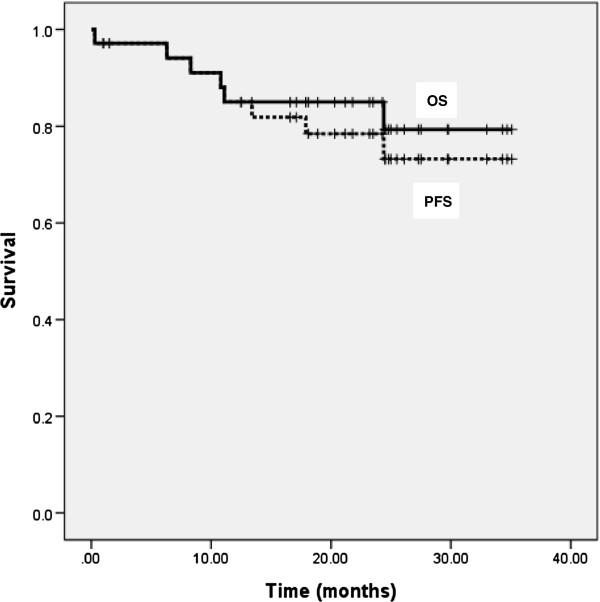
**Kaplan-Meier estimates of progression-free survival (PFS) and overall survival (OS) for all patients.** The 2-year PFS & OS rate was 79.3% (95% CI 58.4 ~ 99.6%) and 73.2% (95% CI 52.4 ~ 93.6%).

After three cycles of induction chemotherapy, 30 patients (85.7%) achieved an objective response (CR in 9 patients, 25.7% [95% CI 11.2–40.2%]; and a partial response [PR] in 21 patients, 60% [95% CI 43.8–76.2%]). Consistent response rates across primary tumor sites were observed in a subgroup analysis and included the oropharynx (CR 17.6%, PR 70.6%), hypopharynx (CR 30%, PR 50%), and larynx (CR 37.5%, PR 50%). After sequential CCRT, 22 patients (62.9%) achieved a CR (95% CI 46.8–78.9%) and eight (22.8%) achieved a PR (95% CI 8.9–36.8%) (Table 2). The mean follow-up duration was 30.3 months. The estimated 2-year PFS and OS rates were 73.2% (95% CI 52.4–93.6%) and 79.3% (95% CI 58.4–99.6%), respectively (Figure 1).

**Table 2:** Response to induction chemotherapy and chemoradiation therapy

	**Primary lesion (n = 35)**		**Lymph node (n = 25)**	
**Response**	**No.**	**%**	**No.**	**%**
After induction chemotherapy				
CR	18	51.4	7	28
PR	11	31.4	13	52
SD	1	2.9	1	4
PD	0	0	0	0
Non-assessable	5	14.2	4	16
Combined response (Primary lesion + LN)				
CR				
No.	9			
%	25.7			
ORR (CR + PR)				
No.	30			
%	85.7			
After Concomitant chemoradiation				
CR	24	68.6	17	68
PR	6	17.1	4	16
SD	0	0	0	0
PD	0	0	0	0
Combined response				
CR				
No.	22			
%	62.8			
ORR (CR + PR)				
No.	30			
%	85.7			

Abbreviations: LN, regional neck lymph nodes; CR, complete response; PR, partial response; SD, stable disease; PD, progressive disease; ORR, overall response rate.

### Quality of life and symptom response

Global QOL was assessed at baseline and at 9 weeks (after 3 cycles of induction chemotherapy) using the EORTC QLQ C-30, version 3. Completed questionnaires were available for 30 patients (Table 3). There was a non-significant decrease in global QOL, from a median score of 56.1 at baseline to 53 at 9 weeks. Emotional functioning was significantly impaired, but other functional QOL scores (*e*.*g*., physical, role, cognitive, and social functioning) showed no significant differences. There were no significant differences in most treatment-related symptoms, except nausea and vomiting. Nausea and vomiting were significantly increased.

**Table 3:** Quality of life scores at baseline and at completion of induction chemotherapy (scored by patients from 0 to 100 using the EORTC QLQ-C30 v.3)

**Functional parameter**	**Scores at baseline**	**Scores after completion of 3 cycles of induction chemotherapy**	***p*value**
Global			
Global health status/QoL	56.1	53	NS
Functional scales			
Physical functioning	84.7	89.7	NS
Role functioning	89	90.9	NS
Emotional functioning	80.2	94.2	< 0.05
Cognitive functioning	85.2	94.2	NS
Social functioning	77.1	83.8	NS
Symptom scales/items			
Fatigue	19.5	24.5	NS
Nausea and vomiting	3.4	9.7	< 0.05
Pain	17.2	10.3	NS
Dyspnea	11.4	11.4	NS
Insomnia	16	16	NS
Appetite loss	17.2	29.8	NS
Constipation	6.8	11.4	NS
Diarrhoea	3.4	10.3	NS
Financial difficulties	26.4	36.7	NS

### Pattern of first relapse

Among 30 patients who showed a complete or partial response after CCRT, five had locoregional recurrence. Four of the five patients showed a PR and one showed a CR after CCRT. All five patients showed initially advanced stage IV disease. Except one patient who showed PD among the patients showing CR, 21 patients had no recurrence until this analysis was done. Among the progressive patients from PR, One patient underwent salvage surgery and chemotherapy, three received only palliative chemotherapy, and one refused additional treatment. Distant relapse was not observed in this study.

### Toxicity

The toxicity of weekly induction TPS chemotherapy was assessed in all 35 patients (Table 4). Grades 3–4 neutropenia occurred in 28.5% of patients, and 8.5% developed febrile neutropenia during induction chemotherapy. The most common grades 3–4 nonhematologic toxicities were diarrhea (17.1%), anorexia/nausea/vomiting (11.4%), and mucositis (8.5%). Anorexia/nausea/vomiting, fatigue/asthenia, mucositis, and diarrhea of less than grade 2 developed in 74.2%, 65.7%, 42.8%, and 42.8% of patients, respectively.

**Table 4:** Acute hematologic and nonhematologic adverse events during induction chemotherapy and concurrent chemoradiotherapy

	**Grade 1-2**		**Grade 3-4**	
	**No.**	**%**	**No.**	**%**
Hematologic				
Neutropenia	9	25.7	10	28.5
Neutropenic fever	0	0	3	8.5
Anemia	22	62.8	1	2.8
Thrombocytopenia	10	28.5	1	2.8
Nonhematologic				
Anorexia/Nausea/Vomiting	26	74.2	4	11.4
Fatigue/Asthenia	23	65.7	1	2.8
Mucositis/Odynophagia	15	42.8	3	8.5
Diarrhea	15	42.8	6	17.1
Neuropathy	4	11.4	0	0
Nephropathy	5	14.2	0	0

Note. Adverse events were graded according to the National Cancer Institute Common Toxicity Criteria, version 3.0.

## Discussion

The TPF regimen remains the standard induction chemotherapy option for patients with advanced HNSCC based on their synergistic effect. Longer overall and progression-free survival and a non-significant reduction in overall toxic effects were evident in the TPF group as compared with PF [17]. However, there was more myelotoxicity in the TPF group (83%) than in the PF group (56%), indicating a clear need for regimens with improved tolerability and lower toxicity.

This study was designed to establish a safe and tolerable outpatient regimen using docetaxel for induction chemotherapy for HNSCC. This study was designed to establish a safe and tolerable outpatient regimen using docetaxel for induction chemotherapy for HNSCC. In a phase I/II trials using 3 weekly TPF in advanced HNSCC, docetaxel was given at a dose of 75 mg/m^2^ every 3 weeks and showed 95% of the rates of grade 3–4 neutropenia and 19% of febrile neutropenia [15]. To reduce the myelosuppresion, Rapidis et al. reported the result using biweekly docetaxel (40 mg/m^2^) with cisplatin and 5-FU, and the rates of grades 3–4 neutropenia was 37% [23]. This results suggested that biweekly or weekly docetaxel could be substituted for 3 weekly regimen if the response are similar. Not only in HNSCC, weekly docetaxel has been studied in variable solid tumors such as gastric cancer, ovarian cancer and lung cancer because of its safety compared with 3 weekly docetaxel regimen [24-28]. In a literature-based meta-analysis of all randomized clinical trials of weekly *versus* three-weekly docetaxel in advanced NSCLC, a significant homogenous advantage in favor of weekly docetaxel was found regarding grades 3–4 neutropenia, with an absolute benefit of 15% to 19%.

In another way to modify 3 weekly TPF regimen there is an increasing trend for the substitution conventional 5-fluorouracil for oral prodrugs, including S-1 and capecitabine, in chemotherapy regimens. S-1 showed response rates of 28.8% to 46.2% with an acceptable toxicit in phase II studies of advanced and recurrent HNSCC [29,30]. Therapy with S-1 plus cisplatin in a phase I/II study was effective and showed acceptable toxicities for advanced/recurrent HNSCC [31]. The S-1/cisplatin combination was also used as induction therapy for advanced HNSCC stage III/IV cancer, and a response rate of 89.7% was reported [32]. To substitute the S-1 for continuous 5-FU infusion in TPF regimen, the studies were performed in advanced gastric cancer patients in advance. According to these trials, the recommended dose for TPS were 60/60/40 (bid) mg·m^2^/d in each [33-35].

Based on these dosese, we conducted the study using TPS for HNSCC. To reduce the hematologic toxicity, we modified the TPS regimen using weekly docetaxel at a dose of 30 mg/m^2^. In a result, the most common hematological and non-hematological toxicities were reduced showing 28.5% of grade 3–4 neutropenia and 17.1% of diarrhea, which were lower than previous results [15,36]. Many patients with advanced HNSCC experience dysphagia from the primary tumor, and difficulty in swallowing capsules containing S-1 may also occur. However, in the present study there was no difficulty or failure in attempts at swallowing S-1 capsules, and mucositis was tolerable. Furthermore, there were no significant changes in most QOL scores as assessed by the EORTC QLQ, except emotional functioning and nausea/vomiting. Recently, one trial of TPS in advanced/recurrent HNSCC have been reported [37]. In this phase I study, they recommended the phase II dose of TPS as 70/70/60 mg·m^2^/d every 3 weeks. However, the rate of grade 3–4 neutropenia was 75% at the recommended dose.

The efficacy of our regimen also showed promise. Patients enrolled in this study had significantly advanced disease; 28 (80%) had stage IV disease, while only seven (20%) had stage III disease. The ORR was 85.7% after induction chemotherapy (CR in 25.7% and PR in 60%) and CR rates increased after CCRT; *i*.*e*., a CR rate of 62.9% and PR rate of 22.8%. These results are comparable to previous reports of induction chemotherapy. In the study by Rapidis *et al*. [15,23], the ORR after induction chemotherapy was 78.1% (CR in 24.4% and PR in 53.7%). Vermorken *et al*. [16] reported an ORR after induction chemotherapy of 68% (CR 8.5% and PR 59.3%). In the present study, all patients who completed the induction chemotherapy (85.7%) showed CR or PR. Therefore, the response rate was 100%. Unexpectedly, five patients dropped out of this study. One patient showed near CR, but he was lost to follow up without response evaluation. Four patients refused further chemotherapy due to grades 3–4 neutropenia or neutropenic fever.

The most common pattern of relapse was locoregional failure without distant metastasis. In fact, four patients who showed recurrence had PR after CCRT; they also had progressive disease during the follow-up period. Only one patient showed definitive recurrence from CR after CCRT. This suggests that a weekly TPF regimen is promising in terms of preventing distant metastasis. Even though radiation was completed after recovery of toxicity during CCRT, radiation interruption may be an explanation of locoregional failure. Cisplatin based CCRT has been a standard regimen for head and neck cancer, however the toxicities are considerable. Based on RTOG 9501 [38], which study compared the benefit of postoperative CCRT *versus* RT, the radiotherapy was delivered in 80% of patients and the planned cisplatin compliance was only 61%. In our study, ten (77%) patients were interrupted the planned RT, but all these patients could receive the planned RT dose after recovery and seven patients showed CR. Among three patients who had PR after CCRT, one patient showed progressive disease during follow up period. Thus a further study aiming to reduce the toxicity during CCRT using cetuximab or weekly cisplatin is warranted.

Even though our data suggest a feasible option for advanced HNSCC, this study has several limitations. First, the higher proportion of patients with oropharyngeal cancer may have affected the response. All 17 patients were smokers, and 12 (70.5%) who had evaluable samples were negative for HPV. Therefore, it seemed that HPV could not influence on the treatment outcome in this study. Second, the dose and number of planned cisplatin treatments during CCRT had to be reduced due to toxicological responses such as mucositis. As mentioned above, an optimal regimen during CCRT should be evaluated after induction chemotherapy. In addition, chronic toxicity was not evaluated because the aim of this study was to determine the efficacy of a weekly TPS regimen. However, a study of chronic toxicity is needed to clarify the effects on the quality of life of long-term survivors.

## Conclusion

In conclusion, a weekly docetaxel, cisplatin, and S-1 combination as an induction regimen showed promising efficacy and safety in locally advanced HNSCC patients. Myelosuppression, which is the most serious and common complication of TPF regimens, was notably low using this regimen. While relatively few patients were included in this study, the results suggest that a weekly TPS regimen represents an alternative to TPF as an outpatient regimen for the treatment of locally advanced HNSCC. Our findings indicate that a phase III trial of the efficacy of this treatment regimen compared to TPF chemotherapy is warranted.

## Competing interest

The authors declare no competing financial interests.

## Authors’ contributions

WKB is a main author. JEH, HJS, KHL, HSH, EKS, HJY, ISC, IJC performed the chemotherapy for patients and revised the manuscript. JKL and SCL performed the operation for patients. WKC performed the radiotherapy for patients and revised the manuscript. SHC conceived of the study, and approved the final manuscript. All authors read and approved the final manuscript.

## Pre-publication history

The pre-publication history for this paper can be accessed here:

http://www.biomedcentral.com/1471-2407/13/102/prepub
